# Complement Defects in Patients with Chronic Rhinosinusitis

**DOI:** 10.1371/journal.pone.0047383

**Published:** 2012-11-07

**Authors:** Maria Q. Gaunsbaek, Bibi Lange, Anette D. Kjeldsen, Viggo Svane-Knudsen, Karsten Skjoedt, Maiken L. Henriksen, Christian Nielsen, Yaseelan Palarasah, Soren Hansen

**Affiliations:** 1 Department of Otorhinolaryngology, Odense University Hospital, Odense, Denmark; 2 Department of Cancer and Inflammation Research, University of Southern Denmark, Odense, Denmark; 3 Department of Clinical Immunology, Odense University Hospital, Odense, Denmark; University of Leicester, United Kingdom

## Abstract

The complement system is an important part of our immune system, and complement defects lead generally to increased susceptibility to infections and autoimmune diseases. We have studied the role of complement activity in relation with chronic rhinosinusitis (CRS), and more specifically studied whether complement defects collectively predispose individuals for CRS or affect CRS severity. The participants comprised 87 CRS patients randomly selected from the general population, and a control group of 150 healthy blood donors. The CRS patients were diagnosed according to the European Position Paper on Rhinosinusitis and nasal Polyps criteria, and severity was evaluated by the Sino-nasal Outcome Test-22. Serum samples were analysed by ELISA for activity of the respective pathways of complement, and subsequently for serum levels of relevant components. We found that the frequency of complement defects was significantly higher among CRS patients than among healthy control subjects. A majority of Mannan-binding lectin deficient CRS patients was observed. The presence of complement defects had no influence on the severity of subjective symptoms. Our studies show that defects in the complement system collectively may play an immunological role related to the development of CRS. However, an association between severity of symptoms and presence of complement defects could not be demonstrated.

## Introduction

Chronic rhinosinusitis (CRS) is a common disease with considerable impact on quality of life and airway morbidity. Phenotyping CRS is still an ongoing subject for discussion and CRS is difficult to diagnose due to the lack of available biomarkers. The causes of CRS are still largely unknown but may involve cilia dysfunction or polymorphism in genes involved in regulation of inflammatory responses [1], [2]. Common underlying disorders such as asthma, allergy and immunodeficiency have been associated with CRS [3]–[5]. In patients with cystic fibrosis the prevalence of CRS is close to 100%. Healthy carriers of a mutation otherwise associated with cystic fibrosis have also a significantly increased prevalence of CRS compared to the general population [6]. Numerous studies support a link between smoking and CRS and several studies describe biofilm formation on sinonasal mucosal surfaces as mediator of the inflammation in CRS [7], [8]. In a Danish study investigating risk factors, an increased CRS prevalence was correlated significantly with occupational exposure to inhaled particles [9]. The mechanisms that underlie inflammation in CRS have not yet been fully revealed.

The complement system is an important part of the innate immune system and helps to clear invading microorganisms. The complement system is activated by three pathways: the classical, the alternative and the lectin pathway. The classical pathway is activated by binding of C1q to antigen-antibody complexes. The lectin pathway is activated by binding of either Mannan-binding lectin (MBL) or Ficolins to microbial surfaces. The alternative pathway is spontaneously and continuously activated in the blood at a low rate by the hydrolysis of the thioester group within C3, but this activation pathway is controlled in the host by several regulatory molecules [10]–[12]. Recently, it was demonstrated that Collectin 11 (CL-11, alias CL-K1) also was associated with complement-activating proteases and may be yet an activator of the lectin pathway [13], [14]. The pathways converge into a common point, when C3 is cleaved into C3a and C3b. Deposition of C3b leads to opsonisation and potentially to formation of a membrane attack complex, C5b-C9, resulting in lysis of microorganisms. During activation, small chemotactic fragments, C5a and C3a, are released to attract and activate inflammatory cells at the site of infection. Albeit there is a large degree of redundancy among the three pathways, it is well known that deficiencies of the complement system can lead to increased susceptibility to infections and inflammatory diseases [11], [15], [16]. Many studies have focused on serious infections and rheumatologic disorders [17]–[20]. Other studies have shown an up-regulation of complement components in human sinonasal tissue of CRS patients [21], [22], indicating that the complement system also plays a role in the sinonasal inflammatory response. Only few studies have focused on the association between complement deficiencies and CRS. Chinese CRS patients were studied by measuring the serum concentration of immunoglobulins, C3, C4 and MBL, but low serum levels of these components were not associated with CRS [23]. In a Finnish study, the levels of immunoglobulins and C4 were analysed, and C4 immunophenotyping was used to detect C4A and C4B deficiencies as null alleles. The study found that C4A null alleles and low IgA and IgG levels were correlated to CRS [24]. We hypothesized that microorganisms are more likely to colonize in sinonasal mucosa in absence of complement activation, and it is reasonable to assume, that deficiencies in the complement system contribute to sustained infections and inflammatory reactions. Alternatively, it is also conceivable that the immune system in absence of sufficient complement activation will be guided in an improper direction. This could lead to unfavourable immune responses against commensal microorganisms with the potential outcome of chronic inflammation. Thus, the study of complement defects in CRS patients is of interest to investigate if CRS is related to inherited deficiencies in the complement system, and to investigate if such impairment is reflected in the degree of symptoms. In contrast with previous CRS-related complement studies, we have focused on randomly selected CRS patients and on activity measurements to cover collectively the whole complement system. We find that complement defects generally seen, are associated with the prevalence of CRS.

## Materials and Methods

### Patients

The Global Allergy and Asthma European Network (GA^2^LEN) is an international network of European Centers of excellence of allergy and asthma. As part of a GA^2^LEN project a postal questionnaire was sent to a representative random sample of 5000 Danish subjects. In a second phase selected respondents – with and without CRS – were invited to a full ear nose and throat examination including rhinoscopy. Respondents who fulfilled the European Position Paper on Rhinosinusitis and nasal Polyps (EP3OS) criteria [25] were diagnosed with CRS. Respondents, who previously had been diagnosed with CRS and received relevant treatment, were also included as CRS patients, although they due to efficient treatment not always fulfilled the EP3OS criteria. All patients diagnosed with CRS were invited for follow-up after 1 and 2 years. At the 2-year follow-up, 88 patients (50 females and 38 males) participated, and serum samples were taken and analyzed for complement activity. To evaluate the degree of symptoms, the Sino-nasal Outcome Test-22 (SNOT-22) [26] was used to divide the severity from the total point score in mild (0–9), moderate (10–29) and severe (>29). The mean age at the time the blood sample was taken was 55 years (range 22–76 years; median age 57 years).

### Ethics Statement

The study was approved by The Regional Scientific Ethical Committee for Southern Denmark.

According to the Danish ethics guidelines all participants provided written informed consent before participating in the study. The study did not imply research outside the country of residence.

### Laboratory methods

Serum samples from the CRS patients were stored at −80 degrees at Odense Patient data Explorative Network. Serum samples were analysed by enzyme-linked immunosorbent assays (ELISA) for functional assessment of individual pathways of the complement system [27]–[29]. In brief, microtiter wells were coated with HSA and incubated with rabbit-anti-HSA (DAKO A001) to form immune complexes, in order to allow binding and activation of C1 in dilution of serum samples. To assess the alternative pathway activity, wells were coated with lipopolysaccharide (LPS, *Salmonelle typhosa/enterica*, ATCC 10749, Sigma cat. L6386). To assess the MBL/Ficolin lectin pathway activities, wells were coated with mannan (Sigma cat. 7024) or acetylated BSA (Sigma, B2518), respectively. To diminish contributions from the alternative and classical pathway, sodium polyanethole sulfonate (Sigma, P2008) was included in a concentration of 0.5 mg/ml in the first dilution of samples. Endpoint measurements for activation of the alternative and the MBL/Ficolin pathways were based on deposition and detection of C3b (clone C3 F1-8) [29]. Endpoint measurements for activation of the classical pathway were based on detection of the membrane attack complex, C5b-C9 (Bioporto, clone DIA 011-0). On all microtiter plates, a pool of 12 sera from healthy individuals served as a serum calibrator defined as 100%. Analyses were carried out in 8 duplicate dilutions and samples with defects were tested additionally to verify the observed defect. Serum samples from 150 healthy blood donors were used as control group for the classical, MBL, and alternative pathway, as described in the previous study by Palarasah and colleagues [29]. Additionally, the Ficolin lectin pathway activity was measured in serum from the same control group during this study. Serum levels of MBL were measured by use of commercial ELISA kit (Bioporto KIT029CE) in accordance with manufacturer's instructions. Serum levels of total IgA were measured on a Binding Site SPAPLUS turbidimetric analyser using the IgA SPAPLUS kit (Binding site group, NK010.S) in accordance with the manufacturer's instructions. Serum levels of CL-11 was measured as previously described [30].

### Statistical analysis

Differences in proportions of complement activity/deficiency between the independent groups, CRS patients (n = 87) *vs*. healthy control group (n = 150), were tested by a chi-squared test. For testing the degree of symptoms in CRS patients with and without complement deficiency a chi-squared test for larger contingency tables was used. Statistical analyses were performed using STATA statistical software, Release 11 (College Station TX, USA).

## Results

### Association between CRS and complement defects

**Figure 1:**
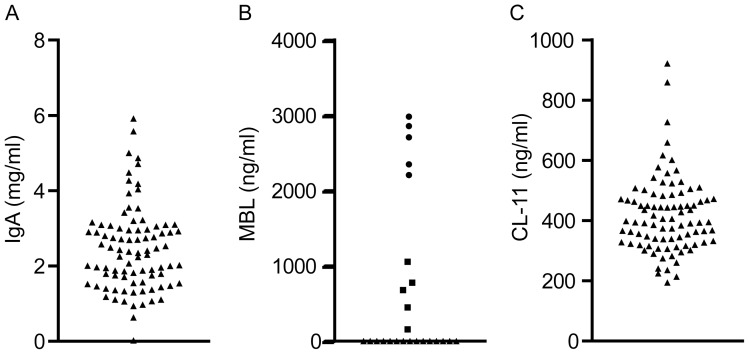
Serum concentrations of IgA, MBL and CL-11. **A**) IgA serum concentration (mg/ml). One CRS patient with a suspected IgA deficiency (<0.021 mg IgA/ml) was identified. **B**) Serum levels of MBL (ng/ml) in the 15 patients with non-detectable MBL pathway activity (triangles). Positive controls illustrated with serum levels of MBL in five genotyped individuals homozygous (circles) or heterozygous (squares) for structural MBL wild type alleles. **C**) Serum levels of CL-11 (ng/ml) among CRS patients. No CL-11 deficient patients were found.

**Figure 2:**
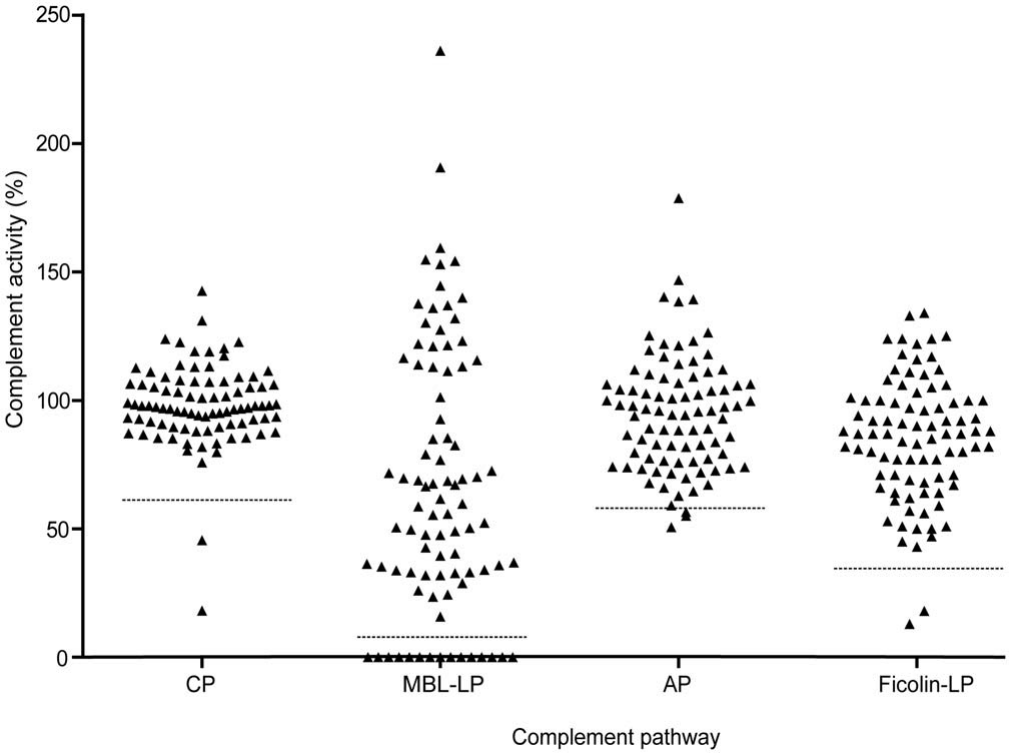
Activity of complement pathways in patients with chronic rhinosinusitis (CRS). The dotted lines indicate the lower cut-off values for normal activity of the given pathway. The cut-off values for the classical, alternative, and Ficolin lectin pathway were defined from the lower limit of a 95% confidence interval. For the MBL lectin pathway the cut-off value was defined as the lowest activity level measured in MBL genotyped donors with at least one structural wild type allele.

**Figure 3:**
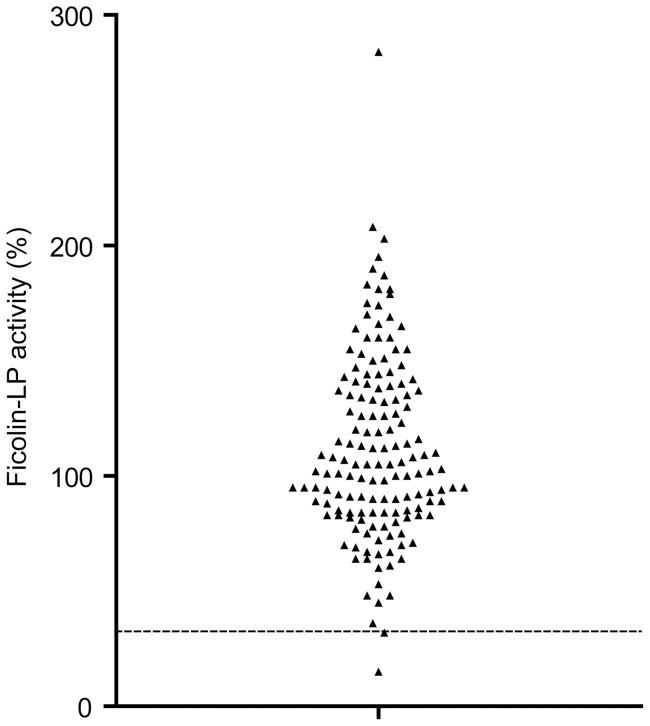
Ficolin lectin pathway activity (%) in the control group of healthy blood donors (n = 150). The dotted line indicates the lower cut-off value for normal activity. The cut-off value for the Ficolin lectin pathway activity was defined from the lower limit of a 95% confidence interval.

**Figure 4:**
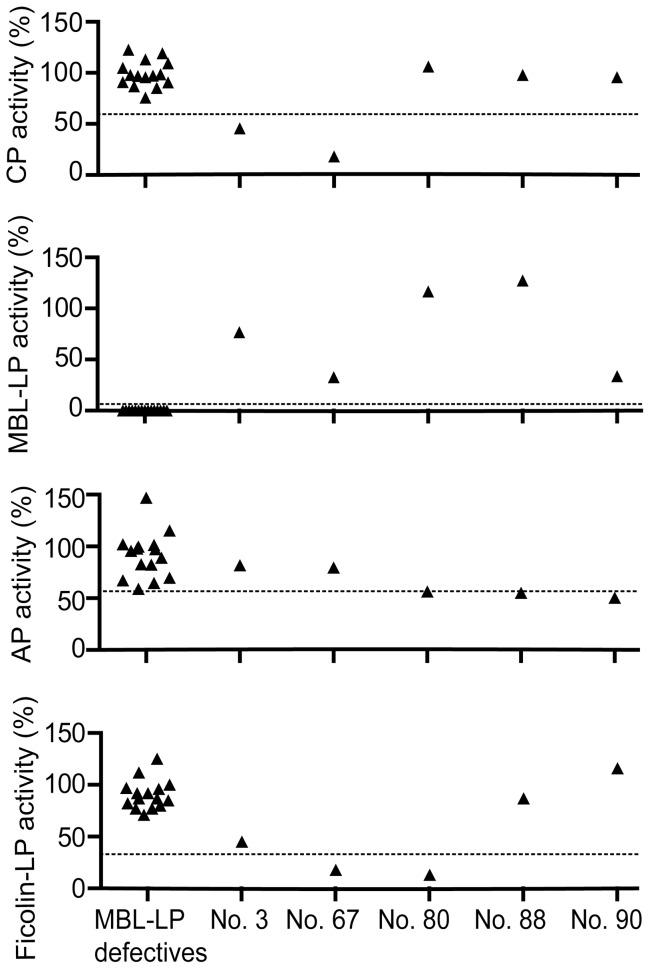
Comparative activity analysis of CRS patients with a potential defect. A lack of functional capacity of the MBL lectin pathway in 15 patients was observed. Patient no. 3 was slightly below the cut-off value in the classical pathway. Patient no. 67 was substantially below the cut-off value in both the classical and Ficolin lectin pathway. The lowest Ficolin lectin pathway activity was measured in patient no. 80, who together with patient no. 88 and 90 had alternative pathway activities marginally below the cut-off value.

IgA deficiency is known to be a strong predisposing factor to CRS, and when we measured the level of IgA in 88 randomly selected CRS patients, we found one CRS patient (no. 57) with undetectable serum IgA <0.21 mg/ml (Fig. 1). To focus the studies on CRS due to complement defects, this patient was excluded in the further analyses, making the total number of patients to be analyzed for complement activity to n = 87. As demonstrated previously the combined activity measurements performed in the current study (Table 1, Fig. 2), allow for detection of multiple variations of complement defects and deficiencies [27], [30]. As reported previously, the complement activity of the classical and alternative pathway followed a normal distribution in the control group of healthy blood donors. The lower limits of relative activity of 95% confidence interval were in the control group 61% and 59% for the classical and alternative pathways, respectively [29]. The distribution of activity of the MBL lectin pathway did not follow a normal distribution. Based on the lowest activity in genotyped healthy individuals with at least one MBL-wildtype structural allele, a lower cut-off activity level was set at 8% [29]. The activities in individuals with homozygous or compound heterozygous structural polymorphisms were 0% [29]. In the control group, the activities of the Ficolin lectin pathway followed a normal distribution, and the lower limit of relative activity of 95% confidence interval was 34% (Fig. 3). When serum samples from the 87 CRS patients were analyzed, we found that 15 (17.2%) showed total lack of MBL lectin pathway activity (Fig. 2). To verify MBL deficiency and exclude MASP-2 deficiency, serum levels of MBL in these patients were measured and found to be below the detection limit of the ELISA (<50 ng/ml) (Fig. 1B). As previously demonstrated this implies that these patients are homozygous or compound heterozygous for polymorphisms leading to structural changes of MBL [29]. Analyzing the activity of the classical pathway, revealed one patient (no. 67, 1.1%) with additionally decreased activity of 18%, which was well below the lower limit of the 95% confidence interval of the healthy control population. Another patient, no. 3, also showed decreased activity of the classical pathway but only marginally decreased activity of the MBL and the Ficolin lectin pathways (Fig. 4). To critically strengthen our observations we considered the patient to have relative overall normal complement activity. Hence, this patient was not included in the group with complement defects. Analyzing the activity of the Ficolin lectin pathway revealed two patients with decreased activity well below the 95% confidence interval (Fig. 2). However, one of these, patient no. 67, was also low in the classical pathway activity (Fig. 4). Only one patient, no. 80 (1.1%), had decreased activity due to Ficolin defects. Although three patients had alternative pathway activity levels below the 95% confidence interval (no. 80, 88, 90), these levels were only minimally decreased, and not considered to be due to major defects influencing the activity of the other pathways (Fig. 4). To critically strengthen our observations patient no. 88 and 90 were considered to have relative normal complement activity and were not included in further laboratory analyses. As a low activity in the Ficolin lectin pathway was seen, patient no. 80 remained in the group of patients with complement defects. No patients were found to be CL-11 deficient (Fig. 1C). In total, we identified 17 CRS patients with complement defects, out of 87 (19.5%). In the control group of healthy persons (n = 150) there were no defectives with decreased activity of the classical and alternative pathway [29]. Within the control group we found one person (0.7%) with a decreased Ficolin lectin pathway activity of 15%. In the control group we found 14 persons (9.3%) with a complete lack of MBL lectin pathway activity that were verified to be homozygous for structural polymorphisms [29]. Thus, collectively the frequency of complement defects was 19.5% in the CRS patient group vs. 10.0% in the control group. As the CRS and control group consist of independent observations, the difference was evaluated by a chi-squared test. The test showed, that the CRS patients had a significantly higher frequency of complement defects in comparison with the control group (p = 0.038). Although the frequency of MBL-deficient individuals in the CRS patients was higher than the frequency in the control group, 17.2% vs. 9.3%, it was not a significant difference (p = 0.073). Based on the relative low number of included persons in the two groups (n = 87 vs. n = 150), we considered the frequency of defectives in the classical and the Ficolin lectin pathway to be too low (1.1%) to allow for separate statistical testing.

**Table 1:** Summary of measurements.

Pathway	Activator	Inhibitor	Endpoint	Additional
CP	IC		C9 (%)	
MBL-LP	Mannan	SPS	C3b (%)	MBL (ng/ml)
AP	LPS		C3b (%)	
Ficolin-LP	AcBSA	SPS	C3b (%)	
				CL-11 (ng/ml)
				IgA (mg/ml)

CP: classical pathway; MBL-LP: mannan-binding lectin pathway; AP: alternative pathway; Ficolin-LP: Ficolin lectin pathway; IC: immune complexes; LPS: lipopolysaccharide; AcBSA: acetylated bovine serum albumin; SPS: sodium polyanethole sulfonate; CL-11: Collectin 11 (alias CL-K1).

### Association between CRS severity and complement defects

The severity of symptoms was divided based on the total SNOT-22 score. The distribution in mild (0–9 points), moderate (10–29 points), and severe (>29 points) was evaluated between CRS patients (Table 2). We found that the distribution of patients among the three severity groups was over all very much alike between CRS patients with or without complement defects. We found by statistical testing no significant difference in severity of symptoms, p = 0.859, between CRS patients with or without complement defects.

**Table 2:** Distribution of severity of symptoms.

Complement defects	Absent No. (%)	Present No. (%)
No. of subjects	70	17
Mild (0–9)	14 (20.0)	4 (23.5)
Moderate (10–29)	34 (48.6)	7 (41.2)
Severe (>29)	22 (31.4)	6 (35.3)

The severity of symptoms was divided based on the total SNOT-22 score.

## Discussion

It is well recognized that the complement system is an important and integral component of the innate immune response, and that defects or deficiencies of complement components may predispose individuals for infections and autoimmune diseases. With focus on CRS, the patient group in the current study was randomly selected from the general population. As the frequency of complement defects was significantly increased among CRS patients compared with controls (19.5 vs. 10.0%, p = 0.038), we found an association between complement defects and CRS. We found no association between complement defects and the severity of CRS. To evaluate complement defects we applied several activity assays to cover all the pathways and components. By this strategy, defects in any of the complement components should in principal be detected, and the assays have previously demonstrated the ability to detect defects or deficiencies of C2, Factor H, Factor I, C1-inhibitor, MBL, and H-Ficolin [27], [29]. In the group of complement defective CRS patients, the majority were MBL defectives (15 out of 17). By studying MBL defects alone, the difference between the CRS group and controls was only close to significant (p = 0.073). The additional two defectives (2 out of 17) had either a combined low classical and Ficolin lectin pathway activity (no. 67) or only low Ficolin activity (no. 80). We find it likely that patient no. 67 suffered from either partial deficiency of C2 or C4, leading to a low activity in both the classical and the Ficolin lectin pathway. The MBL lectin pathway activity of patient no. 67 was 33%. Based on preliminary studies measuring the level of H-ficolin, it is likely that patient no. 80 is partial deficient in H-ficolin (not shown). Low activities of both the Ficolin lectin and the classical pathways were also found in patient no. 3 (45 and 46%, respectively). As the activity of the MBL lectin pathway was close to the median level (77%), patient no. 3 was not included in the group of defectives. Actually, patient no. 3 may also suffer from partial C2 or C4 defect, compensated by a high serum level of MBL (>4 µg/ml, not shown), which is the major determinant in the activity of this pathway. However, inclusion of patient no. 3 in the complement defective group would only have strengthened our findings. Type I C2 deficiency is the most common cause of C2 deficiency and is represented with a frequency of 1∶20,000 in Caucasians [31]. The clinically manifestations vary considerably with presence of rheumatic diseases, increased susceptibility to infections or asymptomatic individuals [20]. Complete C4 deficiency with homozygosity for both C4A null and C4B null alleles is rare. The frequencies of homozygous C4A and C4B null alleles are approximately 1% and 3%, respectively, in a Spanish and UK cohort [32].

Unlike the current study, others have addressed associations between complement defects and CRS by studying single components, and by means of estimations of serum concentrations and genotyping [19], [23], [24]. One can argue that the great redundancy of the complement system where: isoforms overlap in function, number of gene copies varies and pathways merge, and also partly overlap in function makes it difficult to interpret the importance of a given association. Some studies are also hampered by the regulation of single or multiple components, due to inflammatory stimuli, and do not take the eventual presence of neutralizing auto-antibodies into account [21], [23], [33], [34]. Many of these aspects are taken into consideration by looking at activity levels, leading to identification of functional defective individuals. As such, it is the functional integrity, rather than the level of a single component, which is important for association between complement defects and diseases. We aimed at answering the question, whether or not the complement system play a role in either preventing microbial colonization or excessive inflammation leading to CRS. Our finding, that defects in the complement system collectively are associated with CRSs but not with the severity, falls partly in line with the previous finding of association between C4A deficiency and CRS [24]. In the study by Seppänen and colleagues they found a strong association between C4A null alleles and CRS. Another work showed relative high but non-statistically tested occurrence of severe and minor infections – including recurrent sinusitis – in patients with C2 deficiencies [19]. Our findings and partly also Seppänen and colleagues findings, are in contrast with previous work showing lack of association between C3, C4 and MBL deficiencies and CRS [23]. Cui and colleagues' work were mainly based on measurement of serum levels of complement components that may have been influenced by some of the complications stated above. However, corresponding to the findings by Cui and colleagues, we found no significant association between CRS and MBL defects alone. In contrast with previous studies, the patient group in the current study was included randomly from the general population, and selected objects were well examined and diagnosed with CRS based on the latest diagnostic criteria [25]. In other studies, CRS patients were recruited from patients already undergoing treatment or examinations at hospital units [19], [23], [24]. Such patients are likely to present major CRS symptoms with a high degree of severity; and such recruitment differences are likely to play a role with respect to discrepant findings of association between complement defects and severity. However, identifying association based on randomly selected CRS patients strengthen the observed association and is also likely to ensure an objective evaluation of CRS severity. Based on the previous observed strong association between C4A deficiency and CRS [24], it is likely that if we had focused on patients admitting hospital units, would have found even stronger association between complement defects and CRS – potentially also an association with severity. For the majority of patients in current study no defects in complement functional activity were identified, hence there is no evidence for a role of complement in the development of CRS in these patients. What can be concluded is that in a fraction of patients, 17 out 88, complement likely plays a role in the development of CRS. However, in these patients CRS severity appears independent of the complement defects.

In case of recurrent sinus infections or respiratory tract infections in general, it should be considered, whether complement defects exist as an underlying cause. Medical advice and treatment with purified complement components would require identification of the exact cause of functional complement pathway defect. Today, treatment with exogenous supply of active complement components is not yet possible. However, the clinician can prepare patients for repeated antibiotic therapies, and instruct them to be alert for symptoms of serious infections or autoimmune diseases associated with the given complement defect.
